# Cardiac auscultation skills among junior doctors: effects of sound simulation lesson

**DOI:** 10.5116/ijme.5eb6.70c6

**Published:** 2020-05-20

**Authors:** Yasuharu Tokuda, Tetsutaro Matayoshi, Yasunori Nakama, Masaru Kurihara, Tomoharu Suzuki, Yusuke Kitahara, Yuya Kitai, Takashi Nakamura, David Itokazu, Tatsuya Miyazato

**Affiliations:** 1 Muribushi Okinawa Center for Teaching Hospitals; 2 Okinawa Clinical Simulation Center, University of the Ryukyus Hospital; 3 Department of Intensive Care Medicine, Tomishiro Chuo Hospital; 4 Department of Hospital Medicine, Urasoe General Hospital; 5 Department of Emergency and Critical Care Medicine, Urasoe General Hospital; 6 Department of Emergency Medicine, Nakagami Hospital; 7 Okinawa Asia Clinical Investigation Synergy

**Keywords:** Physical examination, cardiac examination, cardiac auscultation, simulation-based medical education, instructional design

## Abstract

**Objectives:**

To evaluate the effect of a sound simulation lesson to improve cardiac auscultation skills among junior doctors.

**Methods:**

This study is based on the design of test comparison before and after educational intervention using a convenient sample. For 50 junior doctors in Japan, diagnostic accuracy before and after a sound simulation lesson for cardiac auscultation skills was compared. There were 15 doctors who experienced cardiology rotation. The lesson used seven abnormal cardiac recordings (third heart sound, double gallop, aortic stenosis, aortic regurgitation, mitral stenosis, mitral regurgitation, and pericardial friction rub). At tests before and after the lesson, the doctors listened to random sound outputs of the same seven recordings, chose diagnostic findings from multiple-choice items, and obtained individual diagnostic accuracy based on the total number of choosing correct findings. Top 10 doctors obtaining the greatest individual accuracy received a commendation.

**Results:**

Pre-lesson diagnostic accuracy was not different between doctors with cardiology rotation training (total diagnostic accuracy of the group, 27/105 [26%]) and those without cardiology rotation (70/245 [29%]). Compared to pre-lesson, post-lesson total diagnostic accuracy significantly improved with about two-folds (97/350 [28%] vs 170/350 [61%]; McNemar Test, p<0.0001). The improvement was significant for double gallop (5/50 [10%] vs. 15/50 [30%]), mitral stenosis (0/50 [0%] vs. 6/50 [12%]), and pericardial friction rub (1/50 [2%] vs. 35/50 [70%]).

**Conclusions:**

The use of a simple sound simulation lesson may help junior doctors to learn cardiac auscultation skills. Clinician educators are encouraged to use this strategy in addition to cardiology rotation training.

## Introduction

Cardiovascular diseases are the leading cause of mortality worldwide, claiming about 20 million deaths annually.^1^ Because its effective treatment depends on accurate and timely diagnosis, it is important to improve the diagnosis not only in cardiology clinics or departments but also in broad clinical settings, including primary care or internal medicine. Technological advancement, such as echocardiography, has contributed to the improvement, but this has led to the relatively low enthusiasm for cardiac physical examination, especially auscultation. In fact, poor cardiac auscultation skills among doctors have been proven.^2^^,^^3^ These skills were unfortunately considered as lost art by some experts.^4^^-^^6^

However, auscultation can immediately identify numerous important cardiac pathologies at little cost and echocardiography may not be available and costly in usual clinical settings.^7^ Thus, it is still important for physicians with specialties such as primary care or internal medicine to learn cardiac auscultation skills. Its training has regained renewed enthusiasm with the assistance of simulation-based learning. A recent meta-analysis and systematic review showed the usefulness of simulation-based training to improve the skills.^8^ However, there is an issue of instructional design about how to effectively provide learning experience among physicians for improving cardiac auscultation skills.

Here we propose an instructional design to enhance the learning of cardiac auscultation skills: pre-test, sound simulation lesson, and post-test. Use of high-quality speaker for producing low-pitch sounds like cardiac sounds or murmurs in a listening lesson can be an effective tool for teaching a large number of learners in a limited time.^9^ In addition, scoring or gamification may facilitate motivation of learning.10 Thus, in the current study, we evaluated whether it would be effective to conduct a sound simulation lesson for learning cardiac auscultation skills.

## Methods

### Study design and participants

The current study was based on comparing tests for diagnostic accuracy before and after the lesson. A convenient sample was used in existing simulation training event which has been held on a Sunday of every summer. This event is intended for junior doctors of first postgraduate year in residency programs in Okinawa. A few doctors of the second postgraduate year participated in the training, and these doctors were also included in the current study with a total of 50 participants. All of them provided informed consent to the study. The ethics of the current study was approved by the institutional review board of the Muribushi Okinawa Center for Teaching Hospitals.

### Sound simulation lesson

The educational intervention was conducted with a sound simulation lesson of seven different abnormal cardiac pathologies (third heart sound, double gallop, aortic stenosis, aortic regurgitation, mitral stenosis, mitral regurgitation, and pericarditis) by a sound/murmur simulator with acoustic speaker capable of output performance of deep, low-pitched sound that mimics the actual cardiac abnormal sounds. The sound output was produced in a single quiet room for all participants to hear. Sound simulation output of each pathology was provided for about two minutes, and thus total lesson time required 14 minutes (2 min times 7 outputs).

### Pre- and post-test

Regarding pre- and post-lesson tests, outputs of seven abnormal cardiac sounds or murmurs were produced in a quiet room. For each output, participants were required to choose correct diagnosis and multiple-choice with 9 sound/murmur items of cardiac abnormal finding included the following: third heart sound, fourth heart sound, systolic click, opening snap, systolic ejection murmur, systolic regurgitant murmur, diastolic regurgitant murmur, diastolic rumble, and pericardial friction rub.

 The following assessment method was based on that by Mangione and colleagues.^2^ The diagnostic accuracy was recognized only when the doctors chose correct items of auscultatory finding. If they chose items of absent finding, this choice was considered as an inaccurate diagnosis. Table 1 shows correct items for each pathology. The top 10 residents obtaining the greatest number of the diagnostic accuracy based on the sum of pre- and post-tests received commendation with medical book prizes (about 20 USD value for each book).

**Table 1:** Sound output pathology and correct auscultatory finding of tests

Output pathology	Correct auscultatory finding
(1) Third heart sound	Third heart sound
(2) Double gallop	Third heart sound and fourth heart sound
(3) Aortic stenosis	Systolic ejection murmur
(4) Aortic regurgitation	Diastolic regurgitant murmur and systolic ejection murmur*
(5) Mitral stenosis	Opening snap and diastolic rumble*
(6) Mitral regurgitation	Systolic regurgitant murmur
(7) Pericarditis	Pericardial friction rub

*Choosing 2 findings are needed for diagnostic accuracy. The sound output order of the pre-lesson test: (1)-(2)-(3)-(4)-(5)-(6)-(7) The sound output order of the post-lesson test: (6)-(1)-(7)-(3)-(4)-(2)-(5)

### Statistical analyses

Regarding the pre-lesson test, between 15 doctors who completed cardiology rotation training and 35 doctors without the rotation, diagnostic accuracy of the groups were compared. Between pre- and post-lesson tests, the diagnostic accuracy of all participants was compared for all tests and for each test. Statistical analyses were performed with Fisher's Exact Test for unpaired data and McNemar Test for paired data. Two-tailed p-value <0.05 was considered as statistical significance. All analyses were conducted by STATA version 14 (College Station, USA).

## Results

Table 2 shows the frequently chosen items for each sound or murmur in the pre-lesson test. The highest diagnostic accuracy was noted in aortic stenosis (correct item: systolic ejection murmur). The most challenging diagnosis was recognized in mitral stenosis (correct items: opening snap plus diastolic rumble).

Total diagnostic accuracy of the pre-lesson test of all participants was 97/350 (28%). Total diagnostic accuracy was 27/105 (26%) among the cardiology rotators and 70/245 (29%) among the cardiology non-rotators. The cardiology rotation training was not associated with greater auscultation accuracy compared with cardiology non-rotation (Fisher’s Exact Test, chi-square (1, N = 350) = 0.299, p=0.61). Total diagnostic accuracy between four postgraduate-year-of-2 junior doctors (8/28 [29%]) and other 46 doctors (89/322 [28%]) were not statistically different (Fisher’s Exact Test, chi-square (1, N = 350) = 0.011, p=0.99).

Table 2. Frequently chosen top three items for each pathology (parenthsis indicates the number of being choisen, N=50)

S3=third heart sound; S4=fourth heart sound; AS=aortic stenosis; AR=aortic regurgitation; MS=mitral stenosis; MR=mitral regurgitation (^*^indicates correct/present item)

**Table 2:** Frequently chosen top three items for each pathology N=50)

Pathology	Most frequently chosen item	2nd	3rd
S3	Opening snap (19)	Systolic click (14)	S3 (11)^*^
Double gallop	S3 (20)^*^	Systolic regurgitant murmur (14)	S4 (11)^*^
AS	Systolic ejection murmur (41)^*^	S3 (3)	S4 (2)
AR	Diastolic regurgitant murmur (19)^*^	Diastolic rumble (11)	Systolic regurgitant murmur (8)
MS	Opening snap (18)^*^	S3 (10)	Pericardial friction rub (6)
MR	Systolic regurgitant murmur (28)^*^	Systolic ejection murmur (14)	Systolic click (10)
Pericarditis	Diastolic rumble (19)	S4 (13)	S3 (12)

S3=third heart sound; S4=fourth heart sound; AS=aortic stenosis; AR=aortic regurgitation; MS=mitral stenosis; MR=mitral regurgitation; (*indicates correct/present item)

Compared to the pre-lesson test, the post-lesson auscultation diagnostic accuracy significantly improved (97/350 [28%] vs 170/350 [61%]; McNemar Test, p<0.001). Diagnosis accuracy of all pathologies increased by the lesson (Table 3). The improvement was statistically significant for double gallop (5/50 [10%] vs. 15/50 [30%]), mitral stenosis (0/50 [0%] vs. 6/50 [12%]), and pericardial friction rub (1/50 [2%] vs. 40/50 [80%]).

**Table 3:** Pre- and Post-lesson diagnostic accuracy (N=50)

Output pathology	Pre-lesson/N (%)	Post-lesson/N (%)	p-value^*^
S3	10 (20)	14 (28)	0.349
Double gallop	5 (10)	15 (30)	0.012
AS	41 (82)	46 (92)	0.137
AR	19 (38)	20 (40)	0.838
MS	0 (0)	6 (12)	0.012
MR	21 (42)	29 (58)	0.110
Pericarditis	1 (2)	40 (80)	<0.001
Total	97/350 (28)	170/350 (61)	<0.001

*Based on McNemar test S3=third heart sound, S4=fourth heart sound, AS=aortic stenosis, AR=aortic regurgitation, MS=mitral stenosis, MR=mitral regurgitation.

## Discussion

Cardiac sound simulation lesson was effective for improving auscultation skills among junior doctors. However, cardiology rotation training was not associated with greater auscultation skills. Teaching auscultation skills for junior doctors during their clinical rotation should be improved since some experts considered the skills as the lost art. A simple sound simulation lesson combined with pre- and post-lesson tests can be used for reviving the skills.

No effect of cardiology rotation training was found on auscultation skills in the current study. Previous studies showed the skills of cardiac auscultation among cardiology fellows were superior to those of general internists, internal medicine junior doctors or medical students.^2^^,^^11^ Moreover, in one of these studies, there was no difference in the skills between general internists, internal medicine junior doctors and medical students.^11^ A rotation training in cardiology by junior doctors in Japan is not mandatory and relatively short (2-4 weeks) even if they would select the rotation. The typical training in the division of cardiology emphasized more on technology interpretations such as echocardiography, cardiac biomarkers and catheterization but less on auscultation skills.^12^ There were few auscultation skill courses provided by cardiology division in most teaching hospitals in Japan.^12^^,^^13^ Thus, although cardiology fellows and faculty have relatively higher individual skills about cardiac auscultation, their rotating junior doctors are likely to have little chance to improve the skills. Since many faculty physicians of the department of general internal medicine in teaching hospitals in Japan are interested in teaching cardiac auscultation for residents,^14^ rotation training in this department may provide precious opportunity to learn cardiac auscultation for junior doctors.^15^

The current study demonstrates the usefulness of skill-testing before and after the lesson for improving cardiac auscultation skills among junior doctors in Japan. Although previous studies showed declining skills of cardiac auscultation over the last three decades, renewed interest in teaching and learning cardiac auscultation has recently emerged by using a simulation-based training.^8^ Because cardiac auscultation skills are considered as clinical art of listening rather than simple knowledge, it is likely not enough to solely read textbooks or to attend lectures for obtaining the skills. Different type of learning strategy should be used, and simulation-based training is the most promising one.^8^^,^^16^ There have been multiple methods providing simulation-based training, including high-fidelity simulator and simple sound output.^17^^,^^18^ However, use of a high-fidelity simulator has disadvantages; only a small number of learners can use it simultaneously and long hours of instruction are needed for completing a large group of residents, and thus instructors may have severe workload. On the other hand, use of simple sound output lesson can provide teaching for a large group and thus instruction time can be reduced.^9^

The skill-testing before and after lesson might have had an effect to help motivate participants to improve their auscultation skills, although this design has never been evaluated in the context of enhancing learning in cardiac auscultation skills. However, this is consistent with previous studies that demonstrated competition or incentives were associated with greater learning in general.^19^ Additionally, game mechanics can be powerfully motivating and is now considered as a novel design to improve learning in several areas of medicine.^10^

At pre-lesson test, the highest diagnostic accuracy was obtained in auscultation of aortic stenosis (systolic ejection murmur), while accuracy was none for mitral stenosis (opening snap plus diastolic rumble). Only one participant had accuracy for pericarditis (pericardial friction rub). These results are consistent with previous studies.^2^^,^^20^ This is likely to reflect relatively easier auscultatory comprehension about systolic murmur and tougher auscultatory identification about diastolic murmur. Interpretation of a rare sound such as pericardial friction rub is difficult, but it can be learnt easily since it is a specific sound which can be differentiated from other sounds.

Our studies may have several limitations. First, the current study was based on the before-and-after comparison, and thus the effect of the lesson might have a bias because of the regression to the mean. Second, we used both the lesson and the tests as an educational strategy. It might be unclear which of these was dominant for the positive effects.

## Conclusions

Among junior doctors, we found a positive effect by using a simple sound simulation lesson for auscultation skills. Postgraduate training should include the sound simulation lesson for educating a large group in a limited time period. Testing before and after lesson may motivate learners.

### Conflict of Interest

The authors declare that they have no conflict of interest.
